# Everybody's Page

**Published:** 1888-11-10

**Authors:** 


### Everybody's Page.

General E. F. Burton, in his recently-published book, "An Indian Olio," says that there is a well-grounded suspicion that many a secret murder by poison is set down as death from snake-bite. It is difficult to explain otherwise the fact that the mortality from that cause is so much greater among men than cattle.

Dr. Gouverneur M. Smith, of New York, while praising the energy of those chemists who have of late years added so many new drugs to the Pharmacopœia, notably in the department of anti-pyretics, says: "It must be remembered that among the originalities of chemists an article may appear as a "Jekyll" on the shelf of the laboratory and as a "Hyde" on the counter of the apothecary.

Apparently medicine and honesty did not go together in the old days, for the potency of some of the medicaments of old superstitions consisted in their being stolen. The negro still cures warts with a stolen piece of fat bacon; and a stolen potato was long believed to be a specific for rheumatism. Perhaps the strangest charm ever invented by the mind of man was the silver ring which cured epilepsy, the merit of which consisted in its being made out of sixpences stolen from the offertory.

What is the best remedy for cramp? Here is a very simple one suggested by Dr. R. W. St. Clair. Let the patient provide himself with a good strong cord and keep it always by him. A long garter—the yard and a-half of good stout knitting that supported the hose of a by-gone age—will serve the purpose well enough. When the spasm comes on, let him wind this cord round the affected part, take an end in each hand, and give them a good sharp pull. It will hurt a little, it is useless if it doesn't, but the cramp will vanish at once.

Dr. Cesare Agostini, writing in *Lo Sperimentale*, expresses the opinion that taking large and continued doses of bromide of potassium is compatible with perfect health. Surely Dr. Agostini made his experiments—we presume he is speaking from experience and not from theory—on specially robust subjects. The general opinion among medical men is that bromide of potassium is a dangerous drug, tending to diminish the nutrition of the brain, and that the effects of its continued use are analogous to those produced by chloral, opium, and alcohol.

Light and air go together, but we are apt to forget that sunlight—not merely the strong and perceptible rays of the sun, but "the light of common day"—is a hygienic agent in itself. Its action is favourable to the functions of the skin, increases the action of the organs of respiration, makes the blood richer and the digestion more active. It is one of the best tonics known; and the people who insist on living with blinds drawn for fear of the glare and heat and fading power of the sun—a view especially favoured by many "good housekeepers"—are unwise in their generation, and sacrifice their constitutions and complexions to their curtains and carpets.

The best remedy for the early stages of phthisis is pure air, and plenty of it. Let it be dry and warm, if that be possible, but above all, let it be pure. Nothing weakens the organism more rapidly, and tends more to make it susceptible to the malign influences of cold and damp, than the habitual respiration of impure air. There can be little doubt that many people who believe they have a hereditary predisposition to consumption develop a mere constitutional delicacy, which, with proper treatment would probably be overcome, into organic disease, by "coddling" themselves in over-heated and badly ventilated rooms, and avoiding a fair amount of air and exercise.

The popular notion that whiskey and ammonia are antidote to snake poison is a blunder. A long course of careful experiments made by Dr. Weir Mitchell, of Philadelphia, proves that neither of these substances has the least effect on the virus. The whiskey cures in cases of snake-bite mostly occur when the patient has received only a small portion of the virus, and would recover by the unaided action of the *vis medicatrix naturae*, though occasionally alcohol may be useful in keeping up the exhausted system while the poison is being eliminated by natural means.

The action of alcohol on the brain and nervous system is anaesthetic and paralysing; its continuous use weakens the intellect and kills the moral sense. These facts have been used as a palliation of crimes committed under the influence of alcohol, and tender-hearted reformers suggest that the poor dear drunkard should not be hanged for murder, nor imprisoned for theft, but taken to a hospital and treated as an invalid. But if any man, of his own free will, partakes of a substance that he knows will render him incapable of calculating the effect of his actions, is he not responsible for that, and is he not to be punished for putting himself in the moral condition that leads to the commission of crime?

All the world is trying to grow cinchona, for with all that can be said of its careless or too-constant use, quinine is one of the most valuable medicines known. At one time a famine of it was threatened by the reckless destruction of the trees in the South American forests, which were the original home of the cinchona bark, but the governments of the various countries where it is found have now undertaken the regulation of its cultivation and exportation, and will take care that trees are planted to replace those that are destroyed. Meanwhile England has succeeded in growing cinchona in India, Holland has planted it in Java, and the Australians have, under the auspices of Baron Ferdinand von Muller, begun to cultivate it in Victoria.

Here is something really good—a method of conducting warfare to which the Peace Society and members of the Quaker persuasion can hardly object. A certain electrician, Mr. Watson by name, proposes to use nitrite of amyl to reduce the crews of hostile ships to a state of incapacity. Shell your enemy with bombs filled with this substance; when these burst the fumes will reduce all whom they reach to a state of unconsciousness. The conquered vessel is then to be towed to shore, the crew handed over to the doctors to be restored to consciousness, and marched comfortably off to imprisonment without a drop of blood being shed. The idea has its charms, but somehow it does not wholly commend itself to the practical mind.

It is well-known that people who have just escaped death by drowning, say that during the last period of their immersion, after the conscious struggle for life has ceased, they recall, with incredible vividness, all the events of their past life. Admiral Sir Francis Beaumont gave a very clear account of his emotions under such circumstances, which is quoted in Harriet Martineau's *Biographical Sketches*. He first thought of the accident which had just taken place, the awkwardness that had produced it, the effect it would have on his father, the manner in which the latter would break it to the rest of the family, and of a thousand other things connected with home. Then his reflections took a wider range, and he recalled his last cruise, a former voyage and shipwreck, his schooldays, with the progress he had made and the time he had misspent in them, even his boyish pursuits and adventures recurred to his mind. And all this took place between his losing consciousness and his being taken out of the water, that is, within a space of two minutes.

